# Hyperoxia in the exercising failing heart: beneficial or detrimental?

**DOI:** 10.1093/ehjopen/oeaf121

**Published:** 2025-09-30

**Authors:** Christos Kourek, Stavros Dimopoulos

**Affiliations:** Clinical Ergospirometry, Exercise & Rehabilitation Laboratory, 1st Critical Care Medicine Department, Evangelismos Hospital, National and Kapodistrian University of Athens, Ypsilantou 45-47, 10676 Athens, Greece; Clinical Ergospirometry, Exercise & Rehabilitation Laboratory, 1st Critical Care Medicine Department, Evangelismos Hospital, National and Kapodistrian University of Athens, Ypsilantou 45-47, 10676 Athens, Greece; Cardiac Surgery ICU, Onassis Cardiac Surgery Center, L.Syggrou 356, 17674 Athens, Greece

**Keywords:** Chronic heart failure, Oxygen therapy, Hyperoxia, Endothelial dysfunction, Microcirculation, Exercise testing

**This correspondence refers to ‘Oxygen supplementation in ambulatory patients with heart failure: a randomized proof-of-concept study’, by M. Tremblay-Gravel *et al.* https://doi.org/10.1093/ehjopen/oeaf074**

**The author's response is available at: https://doi.org/10.1093/ehjopen/oeaf122**

We read with great interest the article by Tremblay-Gravel *et al*.^1^ on the short-term effects of oxygen therapy on the physiological response and symptoms during ambulation in chronic heart failure (CHF) patients, via the use of portable oxygen concentrator. While the authors report some clinical benefits in terms of peripheral oxygen saturation and perceived exertion, there are some physiological and methodological study issues that should be clarified further prior to definite conclusions.

First, the use of supplemental oxygen at 90% fraction of inspired oxygen even during exertion may pose more harm than benefit in CHF patients. High inspired oxygen concentrations have been shown to induce peripheral vasoconstriction, potentially impairing tissue perfusion and increasing afterload, particularly concerning in HF where endothelial function is already compromised. In a previous pilot study from our institute,^2^ we demonstrated that hyperoxia in patients with pulmonary arterial hypertension showed substantial impairments of peripheral muscle microcirculation, as expressed by decreased tissue oxygen consumption rate and delayed reactive hyperaemia time, likely due to increased oxidative stress and evoked peripheral vasoconstriction and endothelial dysfunction. Similar pathophysiological mechanisms may apply in the CHF cohort, particularly those with preserved ejection fraction or pulmonary hypertension (PH), conditions potentially underrepresented in the present study.

Secondly, the heterogeneity of the population raises questions about the applicability of the results. Seven of the 20 patients had left ventricular ejection fraction > 40%, suggesting a mixed CHF population, yet subgroup analysis was not reported. Moreover, the absence of body mass index data precludes evaluation of possible obesity-hypoventilation syndrome, which could confound the impact of oxygen therapy. Notably, two participants experienced significant desaturation during the test, raising concerns about undiagnosed pulmonary conditions such as chronic obstructive pulmonary disease or pre-capillary PH with right-to-left shunting. Indeed, ambulatory oxygen therapy during exercise has demonstrated improvement in exercise capacity and quality of life in fatigue domain among patients with chronic obstructive pulmonary disease. These are precisely the populations where oxygen might be justified, but such cases should not drive general recommendations for CHF patients, where data are still limited regarding its use. Indeed, high-concentration inhaled oxygen has been shown to have significant haemodynamic effects in patients with left ventricular systolic dysfunction and decompensated HF, decreasing cardiac output and heart rate, and increasing systemic vascular resistance.^3^

Moreover, from a statistical standpoint, the small sample size (*n* = 20) and the assumption of normally distributed outcomes require caution. Normality test results were not reported, and given the pilot nature of the study, continuous variables should ideally have been presented as median and interquartile range, not means ± standard deviation. This oversight could affect interpretation of the reported confidence intervals and significance. Additionally, the lack of blinding introduces substantial observer and participant bias, especially for subjective endpoints such as perceived exertion. Even with a crossover design, unblinded investigators may unintentionally encourage participants or over-interpret Borg scale differences. The finding of a 0.75-point difference, while statistically significant, may not meet clinically meaningful thresholds in this population.

In conclusion, we commend the authors for exploring an under-investigated area of the potential use of oxygen therapy during exercise in CHF. However, we urge caution before extrapolating the apparent short-term benefits of oxygen therapy to broader clinical recommendations. The physiological risks of hyperoxia, particularly oxidative endothelial damage, vasoconstriction, and impaired microcirculation, must be carefully weighed against symptomatic improvements. Well-designed, larger, and stratified trials are essential to clarify which subgroups might benefit from ambulatory oxygen therapy in CHF.
